# Downregulation of CDC25C in NPCs Disturbed Cortical Neurogenesis

**DOI:** 10.3390/ijms24021505

**Published:** 2023-01-12

**Authors:** Xiaokun Zhou, Danping Lu, Wenxiang Yi, Dan Xu

**Affiliations:** 1 Fujian Key Laboratory of Molecular Neurology, Institute of Neuroscience, Fujian Medical University, Fuzhou 350005, China; 2 College of Biological Science and Engineering, Institute of Life Sciences, Fuzhou University, Fuzhou 350108, China; 3 College of Life Sciences, Fujian Agriculture and Forestry University, Fuzhou 350002, China; 4 School of Basic Medical Sciences, Fujian Medical University, Fuzhou 350108, China

**Keywords:** CDC25C, neural progenitor cells, proliferation, cell cycle exit, differentiation

## Abstract

Cell division regulators play a vital role in neural progenitor cell (NPC) proliferation and differentiation. Cell division cycle 25C (CDC25C) is a member of the CDC25 family of phosphatases which positively regulate cell division by activating cyclin-dependent protein kinases (CDKs). However, mice with the *Cdc25c* gene knocked out were shown to be viable and lacked the apparent phenotype due to genetic compensation by *Cdc25a* and/or *Cdc25b*. Here, we investigate the function of *Cdc25c* in developing rat brains by knocking down *Cdc25c* in NPCs using in utero electroporation. Our results indicate that *Cdc25c* plays an essential role in maintaining the proliferative state of NPCs during cortical development. The knockdown of *Cdc25c* causes early cell cycle exit and the premature differentiation of NPCs. Our study uncovers a novel role of CDC25C in NPC division and cell fate determination. In addition, our study presents a functional approach to studying the role of genes, which elicit genetic compensation with knockout, in cortical neurogenesis by knocking down in vivo.

## 1. Introduction

Since the cerebral cortex regulates many delicate and complex physiological behaviors, its developmental processes are precisely controlled in time and space. During corticogenesis in mammals (from embryonic day [E] 9.5-E12.5 in mice), highly homogeneous neuroepithelial cells (NECs) transform into neural precursor cells (NPCs), composed of polar apical progenitor cells (APs) and nonpolar basal progenitor cells (BPs). The self-renewal and differentiation of neural stem cells are completed through symmetrical division and asymmetric division, respectively, so as to maintain the number of cells in the NPC pool and generation of neural and glial cells, including neurons, astrocytes, and oligodendrocytes [1,2,3,4]. The proper progression of these development processes relies on the precise control of the balance of proliferation and differentiation in NPCs. Disturbed proliferation and differentiation of NPCs will lead to severe neurodevelopmental disorders, including microcephaly, autism spectrum disorder, and intellectual disability [5,6,7].

The rate of cell cycle progression and the balance between cell cycle re-entry/exit determine the pool of NPCs and the number of neurons [8]. Cell division cycle 25 (CDC25) phosphatases are highly conserved and act as key regulators in eukaryotic cell cycle control. There are three subtypes: CDC25A, CDC25B, and CDC25C. They function in the activation of cell cycle checkpoints and regulate key transitions between the cell cycle phases during normal cell division [9,10,11,12,13,14]. For decades there has been a strong movement toward understanding the roles of CDC25C in tumor progression. In addition, several studies hint at the role of CDC25C during neuronal degeneration. In prion-infected neurons, the expression of CDC25C and its phosphorylated forms (p-CDC25C-Ser198 and p-CDC25C-Ser216) is significantly down-regulated, which affects the re-entry of neurons into the cell cycle and prevents neurons from completing mitosis, leading to neuronal apoptosis and neuronal degeneration [15]. In Alzheimer’s disease, CDK5 directly phosphorylates CDC25C at multiple sites. CDC25C activates CDK4 kinase, leading to neuronal death. At the same time, after the excessive activation of the *BRCA1* gene, a major player in DNA damage, increased levels of p-CDC25C-216Ser may trigger neurons to re-enter the cell cycle, leading to disease [16,17]. However, the specific function of CDC25C under normal brain development is unknown.

Organisms have developed many genetic compensation systems to maintain normal development in the presence of genetic mutations, which were not observed after translational or transcriptional knockdown [18,19,20]. In *Cdc25c* knockout mice, no obvious phenotypic abnormalities were observed, which may be due to the compensation of *Cdc25a* or *Cdc25b* [21,22]. To study the role of CDC25C in brain development, we knock down the *Cdc25C* gene in rat NPCs by performing in utero electroporation (IUE) in embryonic brains. We focus on studying the role of CDC25C in the developing brain because: (i) *Cdc25c* is preferentially expressed in neural progenitors, including apical radial glial (aRG) and basal radial glial (bRG) of the fetal human neocortex [23,24]; (ii) tumor stem cells share a similar genetic mechanism with NPCs [25], and CDC25C has been widely implicated in having a role in human tumors [14]. Our results demonstrate that *Cdc25c* knockdown induces the premature differentiation of NPCs, possibly by regulating the cell cycle exit index in the developing rat brain. Thus, we reveal a novel function of CDC25C in controlling the progression of NPC maintenance, which was not revealed in *Cdc25c* knockout mice.

## 2. Results 

### 2.1. Silencing of CDC25C Altered Cell Distribution in Developing Neocortex

**Figure 1:**
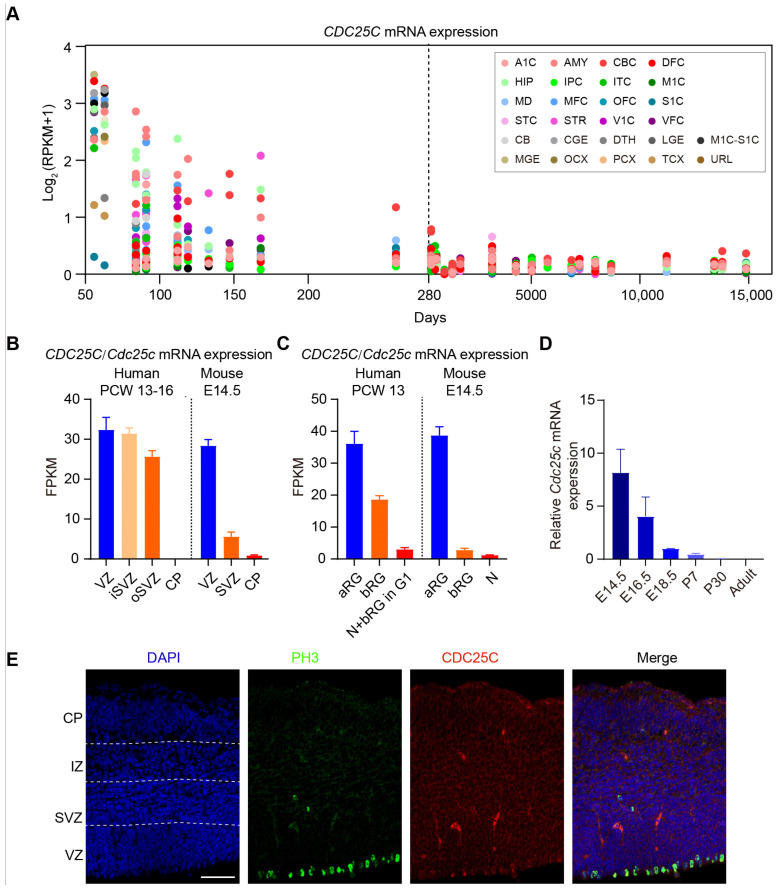
CDC25C is highly expressed in early brain and NPCs. (**A**) mRNA level of *CDC25C* across pre-natal and postnatal periods from BrainSpan data. The X-axis represents the age of samples in days and Y-axis represents the log_2_ (RPKM + 1). The dashed line shows the day of birth. A1C, primary auditory cortex; AMY, amygdaloid complex; CBC, cerebellar cortex; DFC, dorsolateral prefrontal cortex; HIP, hippocampus; IPC, inferior parietal cortex; ITC, inferolateral temporal cortex; M1C, primary motorcortex; MD, mediodorsal nucleus of thalamus; MFC, medial prefrontal cortex; OFC, orbital frontal cortex; S1C, primary somatosensory cortex; STC, superior temporal cortex; STR, striatum; V1C, primary visual cortex; VFC, ventrolateral prefrontal cortex; CB, cerebellum; CGE, caudal ganglionic eminence; DTH, dorsal thalamus; LGE, lateral ganglionic eminence; M1C-S1C, primary motor-sensory cortex; MGE, medial ganglionic eminence; OCX, occipital neocortex; PCX, parietal neocortex; TCX, temporal neocortex; URL, upper rhombic lip. (**B**) Mean FPKM values of *CDC25C/Cdc25c* mRNA expression in the germinal neocortical zones of fetal human neocortical tissue PCW13-16 (n = 6) (left) and embryonic mouse neocortex E14.5 (n = 5) (right). FPKM values are from the dataset GSE38805. VZ, ventricular zone; SVZ, subventricular zone; iSVZ, inner subventricular zone; oSVZ outer subventricular zone; CP, cortical plate. (**C**) Mean FPKM values of *CDC25C/Cdc25c* mRNA expression in indicated isolated cell population of PCW13 fetal human neocortical tissue (left) and E14.5 embryonic mouse neocortex (right). aRG, apical radial glial; bRG, basal radial glial; N, neuron; N + bRG in G1, neuron fraction containing bRG in G1. FPKM values are from the dataset GSE65000. (**D**) mRNA expression of *Cdc25c* in embryonic and postnatal mice brain analyzed by QPCR. E, embryonic day; P, postnatal day. (**E**) E14.5 mouse cerebral cortex were immunostained for CDC25C (red) and phosphorylated histone H3 (PH3, green) antibodies. Nuclei were counterstained with 4′,6-diamidino-2-phenylindole (DAPI, blue). Scale bar 50 μm.

In order to study the role of CDC25C in brain development, we investigated the spatial-temporal expression pattern of CDC25C in the developing brain. First, we analyzed the human developmental brain transcriptome data (Wickramasekara and Stessman, 2019) from BrainSpan (http://www.brainspan.org/ accessed at 2 December 2022). It indicated that *CDC25C* is highly expressed in the early developing brain and shows a sharp decline after birth in the human brain (Figure 1A). Similarly, the *CDC25A* mRNA of different brain regions is high before birth and reduced after birth. However, CDC25B shows comparable expression before and after birth (Figure S1A,B). We then analyzed *CDC25C* mRNA expression in different regions of the human neocortex at 13 to 16 post-conception weeks (PCW) from published transcriptome datasets [23,24]. CDC25C is expressed in the ventricular zone (VZ) as well as the inner and outer sub-ventricular zones (SVZ including iSVZ and oSVZ) but not the cortical plate (CP) in developing human/mouse cortices (Figure 1B). Accordingly, CDC25C mRNA is expressed in the aRG and bRG isolated at PCW 13 of the human neocortex (Figure 1C). Consistently, *Cdc25c* is highly enriched in VZ and aRG in the neocortex of an E14.5 mouse brain (Figure 1B,C). Importantly, *CDC25A*/*Cdc25a* and *CDC25B*/*Cdc25b* showed very similar expression as *CDC25C*/*Cdc25c*, which was enriched in VZ and aRG of developing human and mouse neocortexes (Figure S1C–F). 

We then collected mouse cortex samples at different developmental stages (E14.5, E16.5, E18.5, postnatal day [P]1, P30, and adult) and assayed *Cdc25c* mRNA expression by quantitative real-time PCR. We revealed that *Cdc25c* mRNA was relatively high in the early developmental stage of the cortex and decreased with brain development (Figure 1D). In addition, there was a very low expression of *Cdc25c* in postnatal and adult brains (Figure 1D). We further determined the expression pattern of CDC25C in the mouse embryonic brain by immunohistochemistry. We saw that CDC25C showed cytoplasmic expression and enriched in apical precursors undergoing mitosis at the apical ventricular surface (Figure 1E). Finally, the expression analysis of *Cdc25c* in different tissues of adult mice showed that *Cdc25c* was highly expressed in the testes, spleen, and lung, while the expression level in the whole brain, cortex, hippocampus, heart, and other tissues was extremely low (Figure S2A). Taken together, CDC25C showed high expression in the early brain developmental stage and decreased as the brain developed.

**Figure 2:**
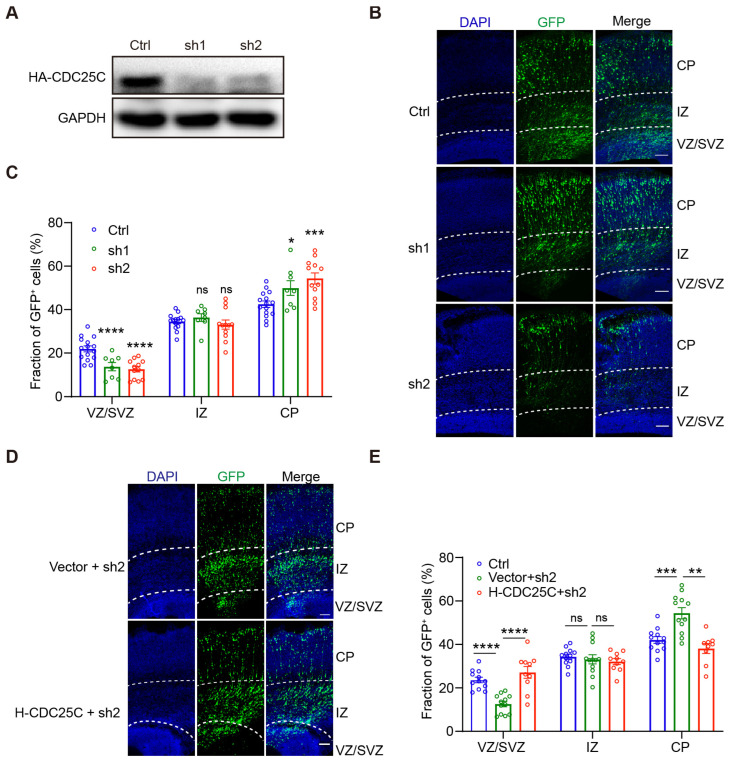
*Cdc25c* knockdown leads to altered cell distribution in the cortex, which can be rescued by human *CDC25C* overexpression. (**A**) HEK293 cells were transfected with scrambled (Ctrl) or *Cdc25c* shRNAs (sh1, sh2) together with mouse *Cdc25c* cDNA; 24 h later, cell lysates were analyzed by immunoblotting with anti-HA antibody, and with GAPDH serving as loading control. (**B**) Coronal sections of rat brains electroporated with Ctrl or *Cdc25c* shRNAs at E16.5 and inspected at E20.5. Slices were stained with DAPI to label nuclei. (**C**) Quantification of cell distribution in (**B**). Data are mean ± SEM. ns *p* > 0.05, * *p* < 0.05, *** *p* < 0.005, **** *p* < 0.0001, one-way ANOVA. Individual data showed brain slice number from Ctrl, n = 7; sh1 and sh2, n = 5 independent mice. (**D**) Coronal sections of rat brains electroporated with *Cdc25c* sh2 together with empty vector or vector expressing human *CDC25C* at E16.5 and inspected at E20.5. (**E**) Quantification of cell distribution in (**D**). Data are mean ± SEM. ns *p* > 0.05, ** *p* < 0.01, *** *p* < 0.005, **** *p* < 0.0001, one-way ANOVA. Individual data showed brain slice number from Ctrl n = 7, vector + sh2, n =3; H-CDC25C + sh2, n = 4 independent mice. Scale bars, 100 μm (**B**,**D**).

Since CDC25C is highly expressed in the developing brain and NPCs, we tried to elucidate the role of CDC25C in brain development by performing IUE in embryonic brains. We first screened small hairpins for *Cdc25c* knockdown capacity. We found two shRNA-expressing vectors targeting different regions of rat/mouse *Cdc25c* mRNA are capable of efficiently downregulating its expression as assayed by Western blot (Figure 2A,B). The scramble control or *Cdc25c* shRNA constructs encoding both GFP were electroporated into NECs in the developing rat cortex at E16.5, and the distribution of the GFP-labeled cells across the cortical zones was examined at E20.5. We found that *Cdc25c* knockdown resulted in significantly fewer cells remaining in the proliferative regions of the VZ/SVZ and significantly more cells reaching the CP compared with the controls (Figure 2B,C). To verify the specificity of *Cdc25c* shRNA, we further co-transfected an shRNA-resistant human-CDC25C construct with *Cdc25c* shRNA2. Our results revealed that human CDC25C could rescue the depletion of GFP-positive (GFP^+^)cells in the VZ and SVZ (Figure 2D,E), thereby excluding the possibility of shRNA off-target effects. These results indicate that *Cdc25c* knockdown disturbs cell distributions in the developing neocortex.

### 2.2. Cdc25c Expression Is Essential for Normal Proliferation and Differentiation of NPCs

**Figure 3:**
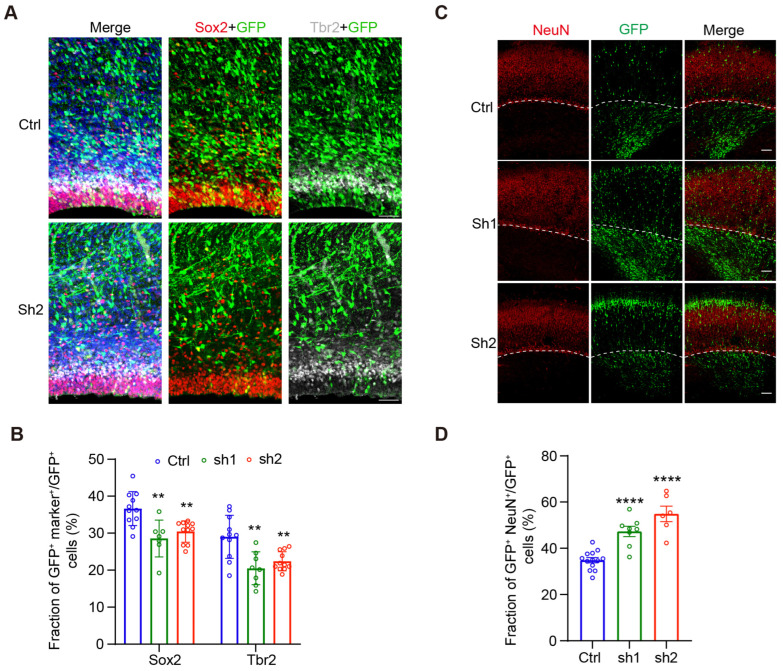
*Cdc25c* knockdown disturb proliferation and differentiation balance of NPCs. (**A**) Coronal Sections of rat brain electroporated with Ctrl or *Cdc25c* shRNAs at E16.5, inspected at E20.5. NPCs in the VZ/SVZ stained for Sox2 (red) and Tbr2 (white) are shown. Scale bar, 50 μm. (**B**) Quantification of NPCs in (**A**). Data are mean ± SEM, ** *p* < 0.01, one-way ANOVA. Individual data showed brain slice number from Ctrl, n = 6; sh1 and sh2, n = 4 independent mice. (**C**) Coronal sections from rat brains electroporated with Ctrl or CDC25C shRNAs at E16.5, inspected at E20.5 and stained for NeuN (red). Scale bar, 100 μm. (**D**) Quantification of cell distribution in (**C**). Data are mean ± SEM, **** *p* < 0.0001, one-way ANOVA. Individual data showed brain slice number from Ctrl, n = 6; sh1, n = 4, sh2, n = 3 independent mice.

Fewer cells remained in VZ/SVZ after *Cdc25c* knockdown, suggesting defects in stem cell maintenance and neuronal differentiation. We examined this possibility by looking at the GFP^+^ cell overlapping with Sox2, a marker of APs, and Tbr2, a marker of BPs. We found a significant reduction in both APs and BPs after *Cdc25c* knockdown (Figure 3A,B). Consistently, we observed a significant increase in GFP^+^ cells overlapping with post-mitotic neurons based on the staining of NeuN (a neuron-specific marker) following *Cdc25c* knockdown (Figure 3C). These results suggest that *Cdc25c* knockdown results in the reduced proliferation and increased differentiation of NPCs.

Since CDC25C is a key protein for cell cycle regulation, we then examined the effect of *Cdc25c* knockdown on the cell cycle. Fluorescent ubiquitination-based cell cycle indicator (FUCCI) is a set of fluorescent probes which enables the visualization of cell cycle progression in living cells [26]. Cultured HEK293 cells were co-transfected with a construct that can specifically label cells in the G1 phase (FUCCI-G1) with *Cdc25c* shRNA or scramble control (Figure S3A). Our results showed that the proportion of G1 cells was significantly increased when transfected with *Cdc25c* shRNA compared with the controls (Figure S3B). In addition, fluorescence-activated cell sorting analysis indicated that CDC25C depletion increased the cell population in the G1 phase, accompanied by a decrease of cells in the S phase (Figure S3C). There was also an increase in the sub-G0 (less than 2N) population in *CDC25C* knockdown cells (Figure S3D), suggestive of cell death. 

**Figure 4:**
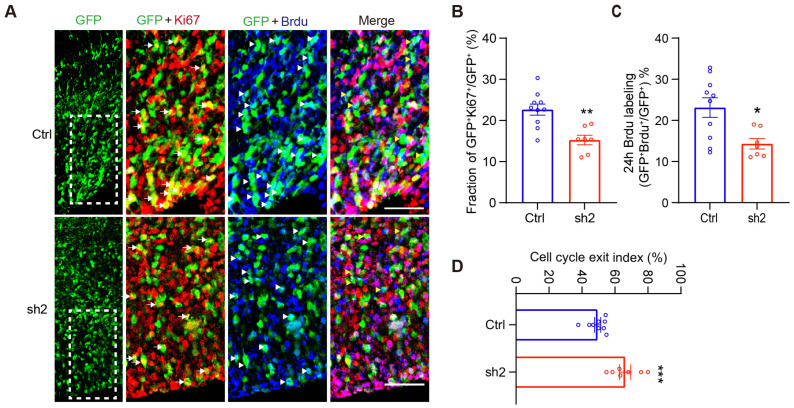
CDC25C is essential for cell cycle regulation. (**A**) Images of the cortices electroporated with Ctrl and *Cdc25c* sh2 at E15.5 and stained for GFP, Ki67, and BrdU at E18.5 (24 h after BrdU labeling). White arrows indicate GFP and Ki67 double-positive cells, white arrowheads indicate GFP and BrdU double-positive cells. Yellow arrowheads indicate GFP and BrdU positive (BrdU^+)^ but Ki67 negative (Ki67^−^) cells. Scale bars, 50 μm. (**B**) Quantification Ki67^+^ cells in GFP^+^ population. Data are mean ± SEM, ** *p* < 0.01, unpaired *t*-test. (**C**) Quantification BrdU^+^ cells in GFP^+^ population. Data are mean ± SEM. * *p* < 0.05, unpaired *t*-test. (**D**) Cell-cycle exit index measured by (BrdU^+^ Ki67^−^)/BrdU^+^. Data are mean ± SEM. *** *p* < 0.001, unpaired *t*-test. (**B**–**D**) Individual data showed brain slice number from Ctrl, n = 4 and sh2, n = 4 independent mice.

To further explore the role of CDC25C in NPC cell cycle regulation, we performed in utero surgery at E15.5, followed by pulse labeling with 5-bromo-2-deoxyuridine (BrdU) at E17.5. The brains were harvested 24 h after BrdU injection (Figure 4A). The quantification of the BrdU/Ki67^−^ positive cells in the GFP labeling population showed a significant decrease of proliferation cells within the *Cdc25c* sh2-expressing samples compared with controls (Figure 4B,C). The cell-cycle exit index was calculated by dividing the number of BrdU^+^ Ki67^−^ cells by the total number of BrdU^+^ cells. We observed a significant increase in cell-cycle exit in *Cdc25c* knockdown cells (Figure 4D). Taken together, our results indicate CDC25C is necessary to maintain the proper cell cycle and proliferation of NPCs. 

## 3. Discussion

CDC25C plays an important role in cell cycle regulation and DNA damage repair. Due to its essential role, higher organisms evolved genetic redundancy. *Cdc25c* knockout mice showed no effect on the biological phenotype. Previous studies have proven that genetic compensation is induced by gene knockouts but not gene knockdowns [18]. We explored the role of CDCD25C in rat brain development by gene knockdown with IUE. *Cdc25c* is highly expressed at the early stage of human brain development and enriched in NPCs. The knockdown of *Cdc25c* results in significantly fewer cells remaining in VZ/SVZ of the cortex, implying NPC reduction. A substantial decrease in Sox2^+^ and Tbr2^+^ cells accompanied by an increase in NeuN^+^ cells revealed the disruption of the balance of NSC proliferation and differentiation. This is further supported by the findings of a significantly increased cell-cycle exit index in *Cdc25c* knockdown NPCs. In the present study, we first indicated that CDC25C played roles in cortical neurogenesis during brain development. 

CDC25A promotes the G1/S transition, and CDC25C is generally thought to function in controlling the G2/M phase transition [14]. In our study, we found that CDC25C depletion in the 293 cell line increased the population of the G1/Go phase cells, accompanied by a decrease in S phase cells. Further study concerning NPCs supported the increased cell cycle exit after *Cdc25c* knockdown. However, whether CDC25C regulates the cell cycle length of NPCs is not known. Further study to analyze the cell cycle duration with BrdU/EdU dual labeling would help. During brain development, NPCs leave the cell cycle and generate neurons that migrate and differentiate to reach their final position in the cortical wall [27]. We note that *Cdc25c* knockdown in NPCs may further impair neuronal migration in the cortical plate. Studies to carefully explore the role of CDC25C in neuronal migration with neuron-specific shRNAs are needed. In addition, whether CDC25C overexpression in NPCs or neurons impairs cortical neurogenesis or neuronal migration should be analyzed further.

Three members of the *Cdc25* gene family are expressed in mammals: *Cdc25a*, *Cdc25b*, and *Cdc25c*. Several studies have addressed the role of *Cdc25a* and *Cdc25b* in neural progenitor development in different models. *Cdc25a* knockdown ameliorates the high mitotic index of *Microcephalin 1*(*Mcph1*)-deficient neuroprogenitors in vivo [28]. Single-cell imaging of the cell cycle reveals that CDC25B over-expression induced heterogeneity of G1 phase length in chick neural progenitor cells [29]. In addition, MCPH1 controls NPC entry into mitosis via the Chk1-Cdc25b centrosome maturation pathway [30]. Our results indicate the role of CDC25C in cortical neurogenesis. However, whether CDC25C takes part in neurogenesis-associated diseases such as microcephaly is unknown. A cell cycle cyclin B1/CDK1 complex is initiated and maintained by CDC25A and CDC25B. However, CDC25C promotes and maintains complete cyclin B1/CDK1 activation, which determines the G2 checkpoint [31]. Double knockout of *Cdc25b* and *Cdc25c* in mice showed normal cell cycle and checkpoint responses [22]. Further study of the roles of different *Cdc25* genes with signal, double or triple knockdown by IUE will help to elucidate the unique role of different genes.

## 4. Materials and Methods

### 4.1. Animals

The female and male Sprague–Dawley rats were bought from Beijing Vital River Laboratory Animal Technology Co., Ltd. (Beijing, China). The rats were bred in the Experimental Animal Center of Fujian Medical University. All animal procedures used in this study were performed according to protocols approved by the Institutional Animal Care and Use Committee of Fujian Medical University. 

### 4.2. Plasmids 

The pCS2+Fucci orange construct was acquired from MBL life science Japan. Mice *Cdc25c* was amplified by PCR from adult mice testis complementary DNA and cloned into the pcDNA3.1-HA vector. Human *CDC25C* was amplified by PCR from 293 cell complementary DNA and cloned into the pcDNA3.1-HA vector. Human CDC25C was synonymously mutated into a form refractory to *Cdc25c* shRNA2 and cloned into pcDNA3.1-HA (from 5′-GTGTTCCTCTGTGAATTCT-3′ to 5′-CGTGTTCCAtTGcGAgTTCTC-3′). Rat *Cdc25c* small hairpin RNAs (shRNAs) were generated in the pLL3.7 vector. The sequence for scrambled (control) shRNA was 5′-TCATGCCTCTATCTACGTC-3′. The targeting regions of *Cdc25c* were as follows: *Cdc25c* shRNA1, 5′-GAAGACCCAATGGAGTGTTC-3′; *Cdc25c* shRNA2 5′-GTGTTCCTCTGTGAATTCT-3′. 

### 4.3. Antibodies

The antibodies used for Western blotting: Mouse GAPDH (Servicebio, Wuhan, China, GB15002, 1:2000) and Rabbit HA (BBI, Shanghai, China, D110004, 1:1000). The antibodies used for immunostaining: Rat BrdU (Abcam, Cambridge, MA, USA, ab6326, 1:1000), Rabbit Ki67 (Abcam, ab15580, 1:1000), Mouse Sox2 (Servicebio, GB14149, 1:1000), Rabbit Tbr2 (Abcam, ab23345, 1:1000), Mouse NeuN (Abcam, ab104224, 1:1000), Rat PH3 (Abcam, ab10543, 1:1000), and Mouse CDC25C (Santa cruz, CA, USA, sc13138).

### 4.4. Cell Culture, Transfection and Western Blotting

Human embryonic kidney (HEK) 293 cells were cultured in DMEM (Hyclone) with 10% fetal bovine serum (FBS) and 1% P/S (penicillin/streptomycin). The HEK293 cells were transfected with Vigofect2000 (Vigorous, Beijing, china) according to the manufacturer’s protocol. Briefly, when the cell density reached 40–60%, the medium was replaced with fresh medium. The DNA was diluted with 0.9% NaCl accordingly, and then the vigoFect transfection reagent was diluted with 0.9% NaCl. It was mixed gently and left standing at room temperature for 5 min. The diluted transfection reagent was added to the diluted plasmid, mixed well, and left standing at room temperature for 15 min. The mixture was added dropwise to the cultured cells. Then, 4–6 h later, the medium was replaced with fresh medium. The cell lysates were prepared 24 h after transfection, and Western blotting was carried out as described previously [32].

### 4.5. RNA Extraction and QPCR Analysis

The total RNA was extracted from the mouse tissue samples using TRIZol reagent (Invitrogen, MA, USA, 15596018). First-strand cDNA was generated using the M-MLV reverse transcriptase (Promega, Madison, WI, USA, M1701) according to the manufacturer’s protocols. Primers for QPCR were as follows: *Cdc25c* Forward: 5′-CTGGCAAGGATTTTCACCAGG-3′, *Cdc25c* Reverse 5′-ATGTGCAGATGTGCTACGCT-3′; *Gapdh* Forward 5′TGATGACATCAAGAAGGTGGTGAAG3′, *Gapdh* Reverse 5′-TCCTTGGAGGCCATGTAGGCCAT-3′.

### 4.6. In Utero Electroporation and BrdU Labeling

Pregnant Sprague–Dawley rats were used. E16.5 embryos from female pregnant SD rats were electroporated as described previously [33]. Briefly, plasmid DNA (2 μg/μL) mixed with 0.1% fast green was injected into the lateral ventricle of the rat embryonic brain with borosilicate glass capillaries. Five pulses, 42 V, 50 ms each, at 950 ms intervals, were delivered for electroporation. For cell-cycle exit experiments, E15.5 electroporated rats were intraperitoneally injected with BrdU (50 mg/kg) at E17.5, sacrificed, and fixed 24 h later. 

### 4.7. Immunostaining

For immunostaining, the brains were fixed in 4% paraformaldehyde in PBS for 24 h at 4 °C, then they were dehydrated in 30% sucrose for 24–48 h and frozen in a tissue-freezing medium. The brains were sectioned into 40 μm and then used for immunostaining. The frozen sections were washed for 5 min with PBS 3 times and then blocked with 10% FBS + 3% BSA in 0.3% Triton X-100 in PBS (blocking buffer) for 1 h at room temperature. The sections were incubated with primary antibodies overnight at 4 °C. They were washed for 15 min with PBST (PBS with 0.3% Triton X-100) 3 times and incubated with secondary antibodies for 1 h, then washed for 15 min with PBST 3 times. Finally, the sections were mounted with the medium. Primary or secondary antibodies were diluted in a blocking buffer. For BrdU staining, the brain slices were treated with 2N HCl for 10 min on ice and 1N HCl for 20 min at 37 °C then immediately neutralized with 0.1 M borate (sodium tetraborate) buffer for 10 min at room temperature; washed 3 times with PBST for 5 min each and then blocked and stained as described.

### 4.8. FACS Analysis

For the FACS analysis, the transfected cells (48 h) were collected and washed with PBS. The pelleted cells were fixed in ice-cold 70% ethanol by adding it with a Pasteur pipette on a vortex. The cells were left at 4 °C for 30 min to a week. The cells were pelleted at approximately 2000 rpm for 5 min and washed twice with PBS. The pelleted cells were washed twice with PBS. Then, 50 μL of RNAse (100 μg/mL, Sigma) was added and incubated at RT or 37 °C for 15 min. Then, 200 μL of propidium iodide was added (50 μg/mL Sigma P4170). A total of 10,000 cells were collected and analyzed by a flow cytometer (BD; FACSCanto II) per sample.

### 4.9. Data Analysis

The sections were imaged on a Nikon A1 confocal microscope. Image quantifications were performed by researchers blinded to the group allocation. The captured images were analyzed, cropped, and brightness and contrast adjusted using Fiji Image J software, Photoshop software and Adobe Illustrator software. The data statistics were analyzed using GraphPad Prism 7 software. All data were mean ± SEM, *t*-test or one-way ANOVA were used for significance analysis. Significant differences between the data were identified by * (*p* < 0.05), ** (*p* < 0.01), and *** (*p* < 0.001), while no significant differences were identified by ns (not significant, *p*> 0.05). Each experiment was performed in at least 3 independent biological replicates to ensure biological replicates.

## Data Availability

All of the data presented in this study are available upon request to the corresponding authors.

## Supplementary Materials

The following supporting information can be downloaded at: https://www.mdpi.com/article/10.3390/ijms24021505/s1.
