# Supplementary material for: Downregulation of CDC25C in NPCs Disturbed Cortical Neurogenesis
Source: Int J Mol Sci. 2023 Jan 12;24(2):1505. doi: 10.3390/ijms24021505 (PMC9863197; doi:10.3390/ijms24021505)
Supplement: Supplementary file 1 [file ijms-24-01505-s001.zip › ijms-2123059-supplementary.pdf]

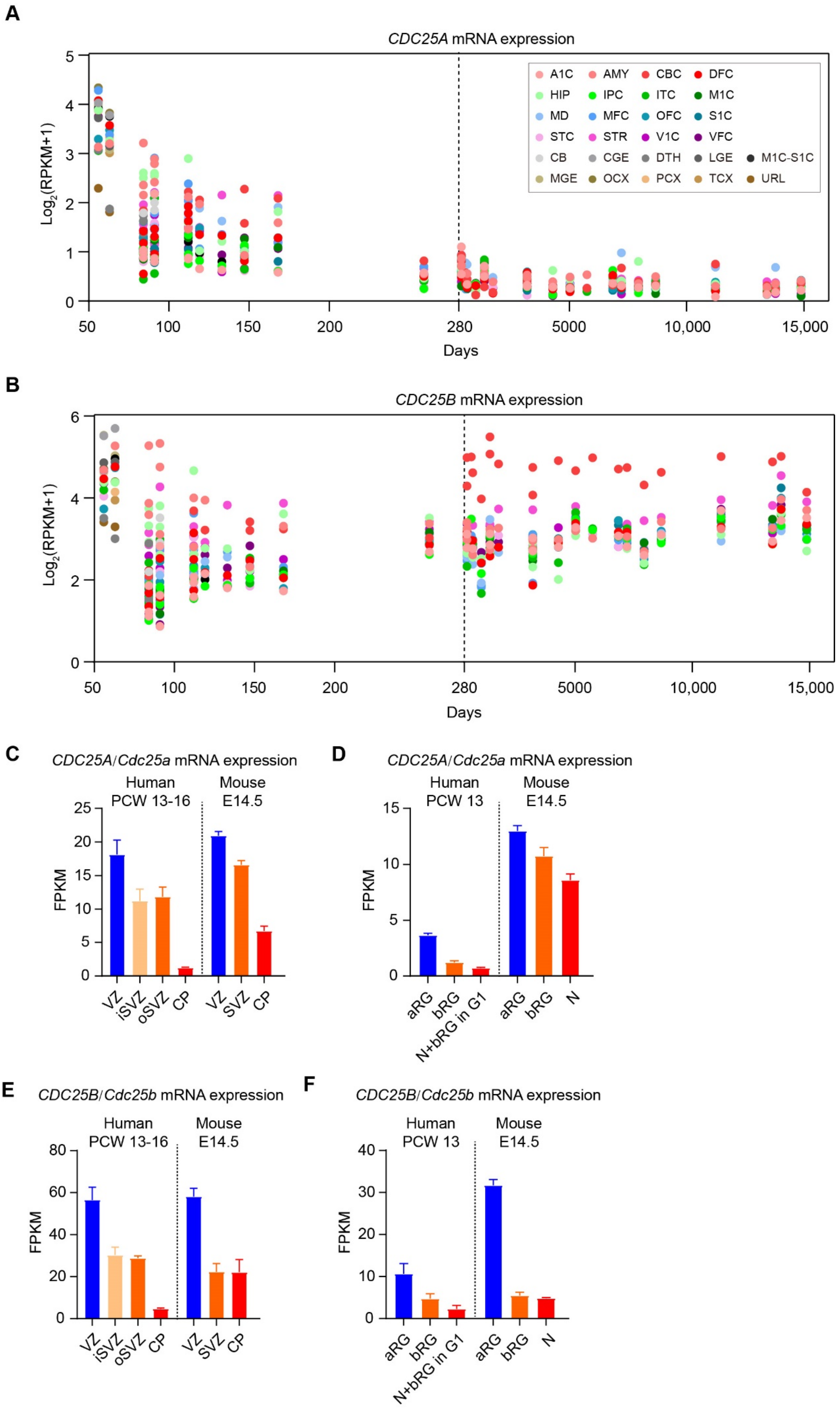
A
CDC25A mRNA expression
Log2(RPKM+1)
A1C AMY CBC DFC
HIP IPC ITC M1C
MD MFC OFC S1C
STC STR V1C VFC
CB CGE DTH LGE M1C-S1C
MGE OCX PCX TCX URL
Days
50 100 150 200 280 5000 10,000 15,000
B
CDC25B mRNA expression
Log2(RPKM+1)
Days
C
CDC25A/Cdc25a mRNA expression
Human PCW 13-16
Mouse E14.5
FPKM
VZ iSVZ oSVZ CP VZ SVZ CP
D
CDC25A/Cdc25a mRNA expression
Human PCW 13
Mouse E14.5
FPKM
aRG bRG N+bRG in G1 aRG bRG N
E
CDC25B/Cdc25b mRNA expression
Human PCW 13-16
Mouse E14.5
FPKM
VZ iSVZ oSVZ CP VZ SVZ CP
F
CDC25B/Cdc25b mRNA expression
Human PCW 13
Mouse E14.5
FPKM
aRG bRG N+bRG in G1 aRG bRG N

Figure S1. Related to Figure 1. (**A**,**B**) mRNA level of *CDC25A* (**A**) or *CDC25B* (**B**) across prenatal and postnatal periods from BrainSpan data. The X-axis represents the age of samples in days and Y-axis represents the log2 (RPKM + 1). The dashed line shows the day of birth. A1C, primary auditory cortex; AMY, amygdaloid complex; CBC, cerebellar cortex; DFC, dorsolateral prefrontal cortex; HIP, hippocampus; IPC, inferior parietal cortex; ITC, inferolateral temporal cortex; M1C, primary motorcortex; MD, mediodorsal nucleus of thalamus; MFC, medial prefrontal cortex; OFC, orbital frontal cortex; S1C, primary somatosensory cortex; STC, superior temporal cortex; STR, striatum; V1C, primary visual cortex; VFC, ventrolateral prefrontal cortex; CB, cerebellum; CGE, caudal ganglionic eminence; DTH, dorsal thalamus; LGE, lateral ganglionic eminence; M1C-S1C, primary motor-sensory cortex; MGE, medial ganglionic eminence; OCX, occipital neocortex; PCX, parietal neocortex; TCX, temporal neocortex; URL, upper rhombic lip. (**C**,**D**) Mean FPKM values of *CDC25A*/*Cdc25a* (**C**) and *CDC25B*/*Cdc25b* (**D**) mRNA expression in the germinal neocortical zones of fetal neocortical tissue PCW13-16 (n = 6) (left) and embryonic mouse neocortex E14.5 (n = 5) (right). FPKM values are from the dataset GSE38805. VZ, ventricular zone; SVZ, subventricular zone; iSVZ, inner subventricular zone; oSVZ outer subventricular zone; CP, cortical plate. (**E**,**F**) Mean FPKM values of *CDC25A*/*Cdc25a* (**E**) and *CDC25B*/*Cdc25b* (**F**) mRNA expression in indicated isolated cell population of PCW13 fetal human neocortical tissue (left) and E14.5 embryonic mouse neocortex (right). aRG, apical radial glial; bRG, basal radial glial; N, neuron; N+bRG in G1, neuron fraction containing bRG in G1. FPKM values are from the dataset GSE65000.

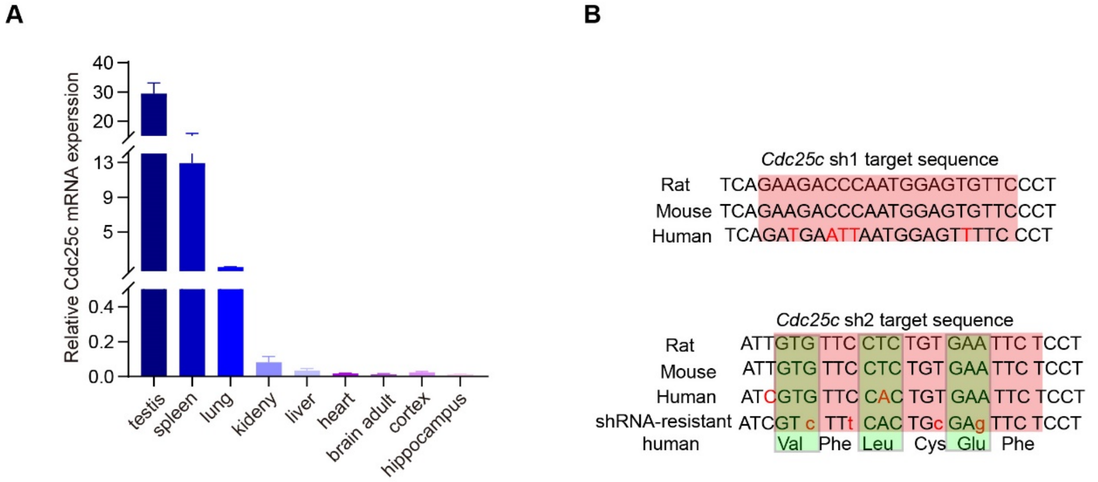

Figure S2. Related to Figure 1 and Figure 2. (**A**) qRT-PCR analysis of *Cdc25c* mRNA in different tissues of adult mice. (**B**) The nucleotide sequences of *Cdc25c* shRNA1 (sh1) and *Cdc25c* shRNA2 (sh2) target sequence in different species.

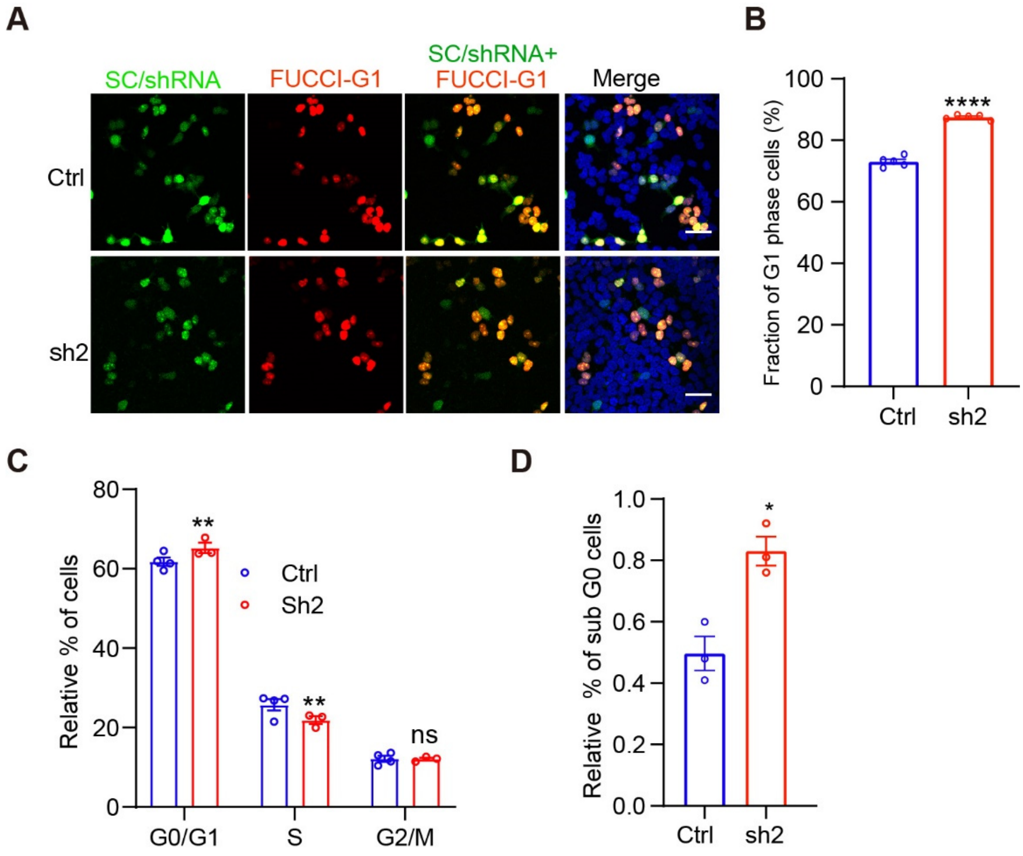

Figure S3. Related to Figure 4. (**A**) HEK293T were transfected with Ctrl or *CDC25C* shRNA2 (sh2) together with FUCCI-G1 fusion protein (Red), 24 h later, cells were fixed and stained with DAPI for nuclei. (**B**) Quantification of G1 phase cells of GFP positive cell in (**A**). Data are mean ± SEM, unpaired *t*-test, **** $p < 0.0001$. (**C**) Quantification of cells transfected with Ctrl or *CDC25C* shRNA2 (sh2) in different cell-cycle phase as analyzed by FACS. (**D**) Quantification of sub Go cells in Ctrl or *CDC25C* shRNA2 (sh2) cells. All Data are mean ± SEM (**C**,**D**). ns, $p > 0.05$, * $p < 0.05$, ** $p < 0.01$, and *** $p < 0.0001$, unpaired s *t*-test. Results are from more than three independent experiments.
